# Supplementary material for: Genetic diversity of SARS-CoV-2 infections in Ghana from 2020-2021
Source: Nat Commun. 2022 May 6;13:2494. doi: 10.1038/s41467-022-30219-5 (PMC9076825; doi:10.1038/s41467-022-30219-5)
Supplement: Supplementary file 1 — Supplementary Information [file 41467_2022_30219_MOESM1_ESM.pdf]

Table of Contents

Supplementary Methods **2**

Sample selection and processing 2

Reverse transcription and Multiplex PCR 2

Library preparation 2

Sequencing 3

Data Analysis 3

Supplementary Tables **4**

Supplementary Table 1: The frequency of all the lineages in 1573 samples sequenced in Ghana from June 2020 to September 2021. 4

Supplementary Table 2: Ghana Population in 2021 (Census) by region and sex, confirmed cases and number of samples sequenced. 5

Supplementary Table 3: Demographic characteristics of community study participants (1002/1123) 6

Supplementary Table 4: Frequency of SARS-CoV-2 variants among travellers arriving in Ghana from other countries in January, March, and June (n=121). 7

Supplementary Table 5: The proportion of Delta sub-lineages (AY) Variants in Ghana. 8

Supplementary Methods

Sample selection and processing

Samples confirmed as SARS-COV-2 positive by Real-Time PCR were selected for genome sequencing. Viral RNA from nasopharyngeal and oropharyngeal samples was extracted using the QIAmp Viral RNA extraction kit (Qiagen, Hilden, Germany). The extracted total RNA concentration was measured using Qubit^{TM} RNA HS Assay Kit on a Qubit 4 Fluorometer (ThermoFisherScientific ^{TM} USA). The integrity and quality of RNA were checked using the Agilent RNA 6000 Nano Kit on the Bioanalyzer (Agilent^{TM} Tech. Inc. CA USA). The ARTIC loCost protocol (https://artic.network/ncov-2019) was used for sequencing as follows with some minor adjustments.

Reverse transcription and Multiplex PCR

The extracted RNA was converted into cDNA using 2µLof the LunaScript® RT SuperMix (New England Biolabs, UK) and 8 µL of RNA. cDNA synthesis reactions were incubated at 25°C for 2 minutes, 55°C for 10 minutes, 95°C for 1 minute and a final hold at 4°C. The ARTIC SARS-CoV-2 Amplicon Panel V3 primers were used for multiplex tiled PCR to generate overlapping amplicons from the cDNA. Two sets of PCR reactions using each primer pool were set as follows; 12.5 µL Q5 Hot Start High-Fidelity 2X Master Mix (New England Biolabs, UK), 4 µL V3 Pool 1 or 2 (10µM) (IDT, USA, cat# 10006788), 6 µL Nuclease-free water and 2.5 µL of the cDNA as template. Cycling conditions for the PCR reaction were set as follows; 98 °C for 30 s, followed by 38 cycles of 98 °C for 15 s and 65 °C for 5 min and a final hold at 4 °C. The PCR products were then run on a gel and only the samples with a visible band were selected for sequencing.

Library preparation

Sequencing libraries were prepared by first combining the amplicons from pools 1 and 2 for each sample and subsequently diluting them at a ratio of 1:10 with nuclease-free water. End preparation was carried out using 3.3 µL of the PCR dilution, 1.2 µL of NEBNext Ultra II End prep reaction buffer, 0.5 µL NEBNext Ultra II End prep enzyme mix (New England Biolabs, UK) and 5 µL nuclease-free water. The mixture was then incubated at 20 °C for 15 minutes, 65 °C for 15 mins and on ice for 1 min. The end prepped Amplicons were then taken through a native barcoding step as follows using either the Ultra II Ligation Module or the Blunt/TA Ligase Master Mix (New England Biolabs, UK); 2.5 µL of the End-preparation reaction mixture, 10 µL of Ultra II Ligation Module master mix (New England Biolabs, UK), 0.3 µL of Ultra II Ligation Module Enhancer (New England Biolabs, UK), 2.5 µL of one of the EXP-NBD196 barcodes was added to each sample (Oxford Nanopore Technologies, UK) and topped up to 20 µL with nuclease-free water. For the Blunt/TA Ligase option, 5µL of Blunt/TA Ligase was added to 1.5 µL of the end-preparation reaction mixture and 1.25 µL of one of the EXP-NBD196 barcodes was added to each sample (Oxford Nanopore Technologies, UK) and finally topped up to 10 µL with Nuclease free water. Barcoding reactions were incubated at 20 °C for 20 minutes, 65 °C for 10 mins and on ice

for 1 min. The barcoded amplicons were then pooled and purified using 0.6X Ampure XP beads (Beckman Coulter) with two 250 µL of SFB (Oxford Nanopore Technologies, UK) washes and a final 200 µL 80% ethanol wash and finally eluted in 35 µL of EB (Oxford Nanopore Technologies, UK). The purified pooled barcoded library was quantified using the Qubit™ DNA HS Assay Kit (Thermo Fisher Scientific, USA) with about 75 ng of barcoded libraries ligated to the AMII sequencing adapters(Oxford Nanopore Technologies, UK) as follows; 5 µL Adapter Mix (AMII) (Oxford Nanopore Technologies, UK), 10µL of NEBNext Quick Ligation Reaction Buffer (5X) and 5 µL of Quick T4 DNA ligase (New England Biolabs, UK) were added to 30 µL of the pooled bar-coded library and incubated at room temperature for 20 mins. The adapter-ligated library was then purified using 0.6X Ampure XP beads (Beckman Coulter) with two 250 µL OF SFB (Oxford Nanopore Technologies, UK) and finally eluted in 15 µL of EB (Oxford Nanopore Technologies, UK).

Sequencing

The purified adapter ligated library was quantified using the QubitTM RNA HS Assay Kit on a Qubit 4 Fluorometer (ThermoFisherScientific TM USA) and about 20 ng was loaded on an R9.4.1 flow cell (FLO-MIN106) for sequencing. The sequencing was carried out using a MinION Mk1b or Mk1c device (Oxford Nanopore Technologies, UK). Our previously published data from March to May 2020 were included in the study to help provide continuity to SARS-Cov-2 genomic epidemiology in Ghana.

Data Analysis

The QGIS Geographic Information System (QGIS software version 3.22.5; http://www.qgis.org) was used to compute the population density (i.e. population size/area[in hectares]) of each region based on the Population and Housing Census data obtained from the Ghana Statistical Service (GSS) (https://www.statsghana.gov.gh). The data was projected on a map downloaded as a shape file from the https://data.gov.gh/dataset/shapefiles-all-districts-ghana-2012-216-districts.

Supplementary Tables

Supplementary Table 1: The frequency of all the lineages in 1573 samples sequenced in Ghana from June 2020 to September 2021.

Variant	Local	Traveller	Total
B.1.617.2 (Delta Lineages)	490	8	498
B.1.1.7 (Alpha)	222	48	270
B.1.1.318	206	4	210
B.1.525 (Eta)	166	15	181
B.1.1	101	7	108
A.27	71	1	72
B.1.1.359	55	1	56
A	30	0	30
B.1	22	5	27
B.1.623	26	0	26
B.1.351 (Beta)	5	7	12
B.1.1.485	9	0	9
B.1.1.409	6	1	7
B.1.1.61	5	0	5
Other Variants	39	23	62
**Grand Total**	**1453**	**120**	**1573**

Supplementary Table 2: Ghana Population in 2021 (Census) by region and sex, confirmed cases and number of samples sequenced.

(https://www.ghs.gov.gh/covid19/dashboardm.php), and (http://www.statsghana.gov.gh)

	Total Population			COVID-19 Cases	
Region	Both Sexes	Male	Female	Cases	Sequenced
**Ghana**	**30,792,608**	**15,182,459**	**15,610,149**	**135,256**	**1,077**
Greater Accra	5,446,237	2,674,192	2,772,045	83,381	465
Ashanti	5,432,485	2,676,394	2,756,091	21,977	47
Eastern	2,917,039	1,434,021	1,483,018	6,795	52
Central	2,859,821	1,390,985	1,468,836	5,289	129
Northern	2,310,943	1,141,708	1,169,235	1,808	55
Western	2,057,225	1,043,400	1,013,825	8,029	91
Volta	1,649,523	786,112	863,411	5,744	103
Upper East	1,301,221	631,258	669,963	1,611	4
Bono	1,208,965	596,841	612,124	2,214	13
Bono East	1,203,306	603,095	600,211	2,817	0
Upper West	904,695	441,799	462,896	805	12
Western North	880,855	451,911	428,944	1,094	0
Oti	747,227	377,380	369,847	890	0
North East	658,903	322,139	336,764	318	0
Savannah	649,627	325,973	323,654	291	0
Ahafo	564,536	285,251	279,285	1,116	0

Supplementary Table 3: Demographic characteristics of community study participants (1002/1123)

Characteristic	N = 1,002[1]
**Sex**	**n (%)**
Female	418 (43%)
Male	561 (57%)
Missing	23
**Age group (%)**	**n (%)**
0-20 Years	104 (11%)
21-40 Years	507 (54%)
41-60 Years	230 (24%)
61-100 Years	106 (11%)
Missing	55
**Sampling Month, Year (%)**	**n (%)**
Mar, 2020	4 (0.4%)
May, 2020	27 (2.7%)
Jun, 2020	9 (0.9%)
Jul, 2020	46 (4.6%)
Aug, 2020	27 (2.7%)
Sep, 2020	18 (1.8%)
Oct, 2020	13 (1.3%)
Nov, 2020	29 (2.9%)
Dec, 2020	27 (2.7%)
Jan, 2021	141 (14%)
Feb, 2021	70 (7.0%)
Mar, 2021	31 (3.1%)
Apr, 2021	60 (6.0%)
May, 2021	96 (9.6%)
Jun, 2021	69 (6.9%)
Jul, 2021	268 (27%)
Aug, 2021	62 (6.2%)
Sep, 2021	5 (0.5%)
**Sampling region**	n **(%)**
Ashanti	47 (4.7%)
Bono East	13 (1.3%)
Central	133 (13%)
Eastern	52 (5.2%)
Greater Accra	490 (49%)
Northern	55 (5.5%)
Upper East	4 (0.4%)
Upper West	12 (1.2%)
Volta	103 (10%)
Western	93 (9.3%)

[1] n (%)

Supplementary Table 4: Frequency of SARS-CoV-2 variants among travellers arriving in Ghana from other countries in January, March, and June (n=121).

Variant	Mar, 2020		Jan, 2021		Mar, 2021		Jun, 2021		Total	
	n	%	n	%	n	%	n	%	n	%
B.1.1.7 (Alpha)			28	42%	19	61%			47	39%
B.1	5	33%	5	8%					10	8%
B.1.1	2	13%	7	11%					9	7%
B.1.617.2 (Delta)							8	89%	8	7%
B.1.351 (Beta)			3	5%	4	13%			7	6%
B.1.525 (Eta)			5	8%					5	4%
A.11	4	27%							4	3%
B.1.1.318					3	10%	1	11%	4	3%
R.1			3	5%					3	2%
A.23.1			1	2%	1	3%			2	2%
B.1.177			2	3%					2	2%
B.1.177.86			2	3%					2	2%
B.1.526 (Iota)					2	6%			2	2%
L.3			2	3%					2	2%
A	1	7%							1	1%
A.21			1	2%					1	1%
B	1	7%							1	1%
B.1.1.10			1	2%					1	1%
B.1.1.409			1	2%					1	1%
B.1.1.420			1	2%					1	1%
B.1.177.7			1	2%					1	1%
B.1.177.81			1	2%					1	1%
B.1.220	1	7%							1	1%
B.1.243			1	2%					1	1%
B.1.36.8			1	2%					1	1%
B.1.617.1 (Kappa)					1	3%			1	1%
B.40	1	7%							1	1%
C.36.3					1	3%			1	1%
**Total**	**15**		**66**		**31**		**9**		**121**	

Supplementary Table 5: The proportion of Delta sub-lineages (AY) Variants in Ghana.

**Delta Plus Variant**	**Count**	**Percentage**
AY.39	174	87%
AY.37	15	8%
AY.5	5	3%
AY.36	2	1%
AY.26	1	1%
AY.30	1	1%
AY.33	1	1%
AY.40	1	1%
Total	200	
