# Supplementary material for: Genetic diversity of SARS-CoV-2 infections in Ghana from 2020-2021
Source: Nat Commun. 2022 May 6;13:2494. doi: 10.1038/s41467-022-30219-5 (PMC9076825; doi:10.1038/s41467-022-30219-5)
Supplement: Supplementary file 3 — Description of Additional Supplementary Files [file 41467_2022_30219_MOESM3_ESM.pdf]

Description of Additional Supplementary Files

File Name: Supplementary Data 1
Description: The Supplementary Data 1 contains Accession numbers for GISAID, GenBank, and European Nucleotide Archive. The sheet names are as follows: (1) GISAID Accessions (2) GenBank Accessions (Ngoi et al) (3) ENA (PRJEB49489) Accessions.
