# Supplementary material for: Genetic diversity of SARS-CoV-2 infections in Ghana from 2020-2021
Source: Nat Commun. 2022 May 6;13:2494. doi: 10.1038/s41467-022-30219-5 (PMC9076825; doi:10.1038/s41467-022-30219-5)
Supplement: Supplementary file 5 — Reporting Summary [file 41467_2022_30219_MOESM5_ESM.pdf]

nature research

Corresponding author(s): Gordon A. Awandare

Last updated by author(s): Feb 17, 2022

Reporting Summary

Nature Research wishes to improve the reproducibility of the work that we publish. This form provides structure for consistency and transparency in reporting. For further information on Nature Research policies, see our Editorial Policies and the Editorial Policy Checklist.

Statistics

For all statistical analyses, confirm that the following items are present in the figure legend, table legend, main text, or Methods section.

n/a	Confirmed	
☐	☒	The exact sample size (*n*) for each experimental group/condition, given as a discrete number and unit of measurement
☐	☒	A statement on whether measurements were taken from distinct samples or whether the same sample was measured repeatedly
☐	☒	The statistical test(s) used AND whether they are one- or two-sided<br>*Only common tests should be described solely by name; describe more complex techniques in the Methods section.*
☒	☐	A description of all covariates tested
☒	☐	A description of any assumptions or corrections, such as tests of normality and adjustment for multiple comparisons
☐	☒	A full description of the statistical parameters including central tendency (e.g. means) or other basic estimates (e.g. regression coefficient) AND variation (e.g. standard deviation) or associated estimates of uncertainty (e.g. confidence intervals)
☐	☒	For null hypothesis testing, the test statistic (e.g. *F*, *t*, *r*) with confidence intervals, effect sizes, degrees of freedom and *P* value noted<br>*Give P values as exact values whenever suitable.*
☒	☐	For Bayesian analysis, information on the choice of priors and Markov chain Monte Carlo settings
☒	☐	For hierarchical and complex designs, identification of the appropriate level for tests and full reporting of outcomes
☒	☐	Estimates of effect sizes (e.g. Cohen's *d*, Pearson's *r*), indicating how they were calculated

Our web collection on statistics for biologists contains articles on many of the points above.

Software and code

Policy information about availability of computer code

Data collection	MinKNOW (MinION software) (version 21.05.25)
Data analysis	All Software used for data analysis are described in the methods section of this manuscript and these include: Guppy (version 3.4.3 -5.0.7), ARTIC MinION (ARTIC version1.2.1), nfcore/viralrecon pipeline (version 2.2), Viral Annotation DefineR (VADR) (version 1.1.3), pangolin-3.1.14, pangoLEARN 2021-10-13, Pango-designation-1.2.86, Nextclade CLI (version 1.4.0), R Statistical Software (version 4.0.4), QGIS software (version 3.22.5), and Nextstrain pipelines (v11). The scripts used for the analysis reported in this study are publicly available at https://github.com/misita-falcon/SARS-CoV-2-Manuscript-2021(https://doi.org/10.5281/zenodo.6375851).

For manuscripts utilizing custom algorithms or software that are central to the research but not yet described in published literature, software must be made available to editors and reviewers. We strongly encourage code deposition in a community repository (e.g. GitHub). See the Nature Research guidelines for submitting code & software for further information.

Data

Policy information about availability of data

All manuscripts must include a data availability statement. This statement should provide the following information, where applicable:

- Accession codes, unique identifiers, or web links for publicly available datasets
- A list of figures that have associated raw data
- A description of any restrictions on data availability

The raw sequencing data (n=1077) is available on the European Nucleotide Archive (PRJEB49489 = (https://www.ebi.ac.uk/ena/browser/view/PRJEB49489?show=reads). Our previous data (n=46) is also available on GenBank with accession numbers ranging from MT890204 - MT890249. The sequences and basic metadata information such as age, gender, location and sample collection date, is freely available at GISAID (https://www.gisaid.org). All accession numbers for GISAID, GenBank, and European Nucleotide Archive are provided in Supplementary Data 1. The summary/analysis tables used to generate the piecharts/stacked

plots are available at https://github.com/misita-falcon/SARS-CoV-2-Manuscript-2021 (https://doi.org/10.5281/zenodo.6375851). The WHO dataset is available on the WHO website (https://covid19.who.int/WHO-COVID-19-global-data.csv). The reference genomes (GenBank number: MN908947.3 and LR757998.1), were obtained from Nextrain Github page (https://github.com/nextstrain/ncov/blob/master/data/references_sequences.fasta) but can also be obtained from GenBank (https://www.ncbi.nlm.nih.gov/nuccore/MN908947.3, https://www.ncbi.nlm.nih.gov/nuccore/LR757998.1). Population and Housing Census data was obtained from the Ghana Statistical Service (GSS) (https://www.statsghana.gov.gh) and the map was downloaded from the Ghana Open Data Initiative (https://data.gov.gh/dataset/shapefiles-all-districts-ghana-2012-216-districts).

Field-specific reporting

Please select the one below that is the best fit for your research. If you are not sure, read the appropriate sections before making your selection.

☒ Life sciences ☐ Behavioural & social sciences ☐ Ecological, evolutionary & environmental sciences

For a reference copy of the document with all sections, see nature.com/documents/nr-reporting-summary-flat.pdf

Life sciences study design

All studies must disclose on these points even when the disclosure is negative.

Sample size	The study is based on data from 1123 viral genomes collected in Ghana during the time period from March 2020 to September 2021. This is an observational study, therefore no sample size calculation is applicable.
Data exclusions	No data was excluded in the analysis.
Replication	This is an observational study based on the samples collected from different health facilities across Ghana during the COVID-19 Pandemic, therefore no replication is applicable.
Randomization	This is an observational study based on the samples collected from different health facilities across Ghana during the COVID-19 Pandemic, therefore no randomization is applicable.
Blinding	This is an observational study based on the samples collected from different health facilities across Ghana during the COVID-19 Pandemic, therefore no blinding is applicable.

Reporting for specific materials, systems and methods

We require information from authors about some types of materials, experimental systems and methods used in many studies. Here, indicate whether each material, system or method listed is relevant to your study. If you are not sure if a list item applies to your research, read the appropriate section before selecting a response.

Materials & experimental systems

n/a	Involved in the study	
☒	☐	Antibodies
☒	☐	Eukaryotic cell lines
☒	☐	Palaeontology and archaeology
☒	☐	Animals and other organisms
☐	☒	Human research participants
☒	☐	Clinical data
☒	☐	Dual use research of concern

Methods

n/a	Involved in the study	
☒	☐	ChIP-seq
☒	☐	Flow cytometry
☒	☐	MRI-based neuroimaging

Human research participants

Policy information about studies involving human research participants

Population characteristics	All patients were confirmed for SARS-CoV-2 infection through RT-qPCR analysis performed on nasopharyngeal swabs. Demographic data was collected including age, sex, date of collection and sampling location. There were more males (57 %, n=561) compared to females (43 %, n=418) among the community participants (n=1002). Most of these community participants (n=1002) were between 21 - 40 years (54 %, n=507) as compared to the other categories; < 20 (11 %, n=104), 41 - 60 (24 %, n=230), and 61 + (11 %, n=106) years (Supplementary Table 3).
Recruitment	Samples were obtained from individuals reporting to community COVID-19 testing laboratories in different regions (Ashanti, Bono East, Central, Eastern, Greater Accra, Northern, Upper East, Upper West, Volta (both Volta and Oti) and Western) of the country, and travellers who tested positive for COVID-19 on arrival at the Kotoka International Airport. Individuals tested at the community laboratories included; patients reporting to hospitals, or those taking tests for travel purposes.
Ethics oversight	The study was approved by the Ethics Review Committee of Ghana Health Service (GHS-ERC 005/06/20), the Ethical

Ethics oversight	Committee of the College of Basic and Applied Sciences of the University of Ghana (ECBAS 063/19-20) and the Research Ethics Committee (REC) of the University of Health and Allied Sciences with certificate number UHAS-REC WV [1] 21-22.

Note that full information on the approval of the study protocol must also be provided in the manuscript.
